# Investigation of the crystallization process of CSD-ErBCO on IBAD-substrate via DSD approach

**DOI:** 10.1038/s41598-020-76848-y

**Published:** 2020-11-17

**Authors:** R. Hayasaka, P. Cayado, M. Erbe, W. Freitag, J. Hänisch, B. Holzapfel, S. Ito, H. Hashizume

**Affiliations:** 1 Graduate School of Engineering, Department of Quantum Science and Energy Engineering, Tohoku University, Aramaki-Aza-Aoba 6-6-01-2, Aoba-ku, Sendai, Miyagi 980-8579 Japan; 2 Karlsruhe Institute of Technology (KIT), Institute for Technical Physics (ITEP), Hermann-von-Helmholtz-Platz 1, 76344 Eggenstein-Leopoldshafen, Germany

**Keywords:** Electronic devices, Chemical engineering

## Abstract

*RE*Ba_2_Cu_3_O_7-δ_ (*RE*BCO, *RE*: rare earth, such as Y and Gd) compounds have been extensively studied as a superconducting layer in coated conductors. Although ErBCO potentially has better superconducting properties than YBCO and GdBCO, little research has been made on it, especially in chemical solution deposition (CSD). In this work, ErBCO films were deposited on IBAD (ion-beam-assisted-deposition) substrates by CSD with low-fluorine solutions. The crystallization process was optimized to achieve the highest self-field critical current density (*J*_c_) at 77 K. Commonly, for the investigation of a CSD process involving numerous process factors, one factor is changed keeping the others constant, requiring much time and cost. For more efficient investigation, this study adopted a novel design-of-experiment technique, definitive screening design (DSD), for the first time in CSD process. Two different types of solutions containing Er-propionate or Er-acetate were used to make two types of samples, Er-P and Er-A, respectively. Within the investigated range, we found that crystallization temperature, dew point, and oxygen partial pressure play a key role in Er-P, while the former two factors are significant for Er-A. DSD revealed these significant factors among six process factors with only 14 trials. Moreover, the DSD approach allowed us to create models that predict *J*_c_ accurately. These models revealed the optimum conditions giving the highest *J*_c_ values of 3.6 MA/cm^2^ for Er-P and 3.0 MA/cm^2^ for Er-A. These results indicate that DSD is an attractive approach to optimize CSD process.

## Introduction

ErBa_2_Cu_3_O_7-δ_ (ErBCO) is one of the *RE*Ba_2_Cu_3_O_7-δ_ (*RE*BCO, *RE*: rare earth) compounds with potential as a functional superconducting layer in coated conductors^1,2^. Indeed, Yoshida et al*.* have demonstrated ErBCO coated conductor samples of nearly 100 m length with average critical current values, *I*_c_, of ~ 700 A/cm-width^3^. Most of the film studies on ErBCO were done via PLD^4^ (pulsed laser deposition) especially regarding possible enhancement of the critical current density, *J*_c_, by perovskite nanoparticles^5^ and nanorods^6^. Just occasional ErBCO film studies are reported for other vacuum (MOCVD^7^, sputtering^8^) and non-vacuum methods (CSD/MOD (metal–organic deposition)^9,10^, sol–gel^11^). Since the ion sizes of Er^3+^ and Y^3+^ are very similar, the stability and growth temperatures of ErBCO and YBCO are well comparable^12^, differing by only ~  ± 10 °C. In this work, since few research has been made on ErBCO CSD process, we prepared ErBCO films on technical IBAD templates^13^ by CSD^14,15^, following the TFA-MOD (metal–organic deposition of trifluoroacetates) route with low-fluorine solutions^16,17^.

In order to find the optimum process conditions for complicated systems such as CSD, one has to deal with numerous parameters, requiring a lot of time and cost for try and error. In particular, the thermal processes involved in CSD have conventionally been optimized in one-factor-at-a-time experiments, i.e., only one of the potentially important parameters is changed, keeping all the others constant. This kind of investigation is extensive and often incomplete because significant parameters, i.e., those influencing and determining the final quality of the films, are often correlated. For example, in CSD, the optimum crystallization temperatures are reported to depend on the oxygen partial pressure^18^. The one-factor-at-a-time approach often fails to find such interactions and requires many experiments to improve process conditions. Therefore, we adopted a novel design-of-experiment (DOE) technique, Definitive Screening Design (DSD)^19^ for the first time, to identify significant factors and improve self-field critical current density, ${\varvec{J}}_{{\mathbf{c}}}^{{{\mathbf{sf}}}}$, at 77 K while reducing the number of necessary experiments as much as possible.

DSD was introduced by Jones and Nachtsheim in 2011^19^. It offers the opportunity to investigate many parameters and optimize a process by performing one experiment with a small number of trials^20,21^. Among the multitude of possible combinations of levels (magnitudes) of the preselected parameters (called “factors”), DSD identifies the few trials to be performed for efficient evaluation of the factors’ effects. Main effects (the first-order effect of a single factor), two-factor interactions (the correlation between two factors), and quadratic effects are estimable at the same time. This feature distinguishes DSD from other, conventional DOE techniques, most of which cannot estimate quadratic effects or need many trials to estimate them. This advantage of DSD makes it possible to find the optimum condition in a large experimental space. By using DSD, one can understand how the target value is changed by the levels of the factors, identify important factors, and finally optimize their levels.

## Experimental

### Solution and sample preparation

The low-fluorine solutions of this study are prepared by mixing fluorinated and non-fluorinated precursor salts, namely Er-propionate or Er-acetate, Ba-TFA, and Cu-propionate, in the stoichiometric ratio Er:Ba:Cu = 1:2:3 in anhydrous methanol resulting in a concentration of 1.5 M (sum of metals). Two types of ErBCO samples were prepared from two different solutions depending on the Er precursor salt (“Er-P” samples from Er-propionate and “Er-A” samples from Er-acetate). The solutions were deposited on 10 × 10 mm^2^ IBAD substrates by spin coating for 30 s at different rotation speeds (2000–4000 rpm). The SuperOx IBAD substrates had the architecture of CeO_2_/LaMnO_3_ (LMO)/MgO/Y_2_O_3_/Al_2_O_3_/Hastelloy C276. The details of the standard pyrolysis and crystallization steps are available in Ref.^22^. The investigated factors were crystallization temperature (*T*_crys_), oxygen partial pressure (*p*_oxy_), dew point (*T*_Dew_), heating ramp, dwell time, and rotation speed. The investigated ranges of these factors and levels are listed in Table 1. We have chosen these parameters because they are known by experience to be the most important parameters that affect the film growth and are directly controllable with our equipment (furnace and spin coater). Other factors might be considered; however, from our experience, we can assume there are no factors that correlate with the six factors in this experiment. That is, these six factors do not have to be considered with other possible factors at the same time.

**Table 1:** Investigated factors and their levels.

Level (coded units)	*T*_crys_ (°C)	*p*_oxy_ (ppm)	*T*_Dew_ (°C)	Dewll time (min)	Heating ramp (°C/min)	Rotation speed (rpm)
Low (−1)	770	150	16	38	10	2000
Medium (0)	780	225	19	64	15	3000
High (1)	790	300	22	90	20	4000

### Thin-film characterization

**Figure 1:**
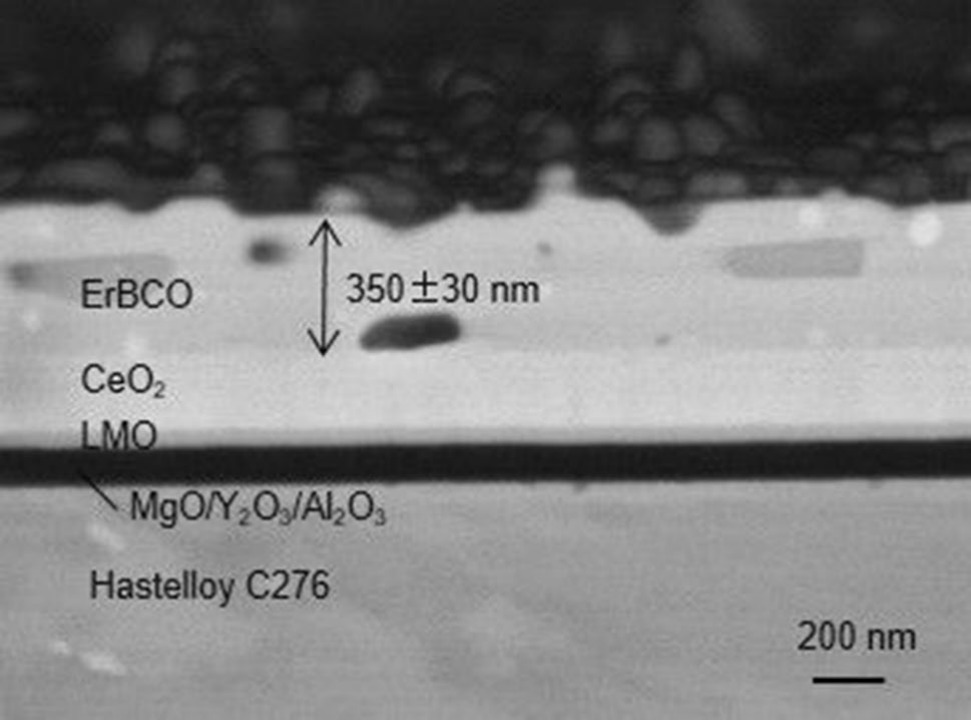
The cross-section of an Er-P sample deposited with 3000 rpm. Buffer layers of MgO/Y_2_O_3_/Al_2_O_3_ delaminated in the course of the cross-section preparation.

The film thicknesses for the rotation speeds of 2000, 3000, and 4000 rpm were 400, 350, and 285 nm, respectively, as analyzed with cross-sectional scanning electron microscopy by a LEO 1530 scanning electron microscope (SEM) with field emission gun (0.1 kV and 30 kV) by *Zeiss*. Figure 1 shows an example of the cross-section of an Er-P sample deposited with 3000 rpm, and the thickness of ErBCO is 350 ± 30 nm. The variation of the thickness is about 10% of the total thickness. Self-field *J*_c_ (${\text{J}}_{\text{c}}^{\text{sf}}$) at 77 K was measured inductively with a Cryoscan (Theva, 50 µV criterion).

### Definitive screening design and model selection

The design matrix for the investigated factors, Table 2, is generated by using a so-called conference matrix^23^. Coded units (– 1, 0, and 1) correspond to the levels in Table 1. The trials (runs) are carried out using the parameter sets (rows) specified in Table 2 in random order. The minimum number of runs is 2*K* + 1 = 13 (*K* is the number of factors), but the runs with level 0 of all factors (center run) was repeated, see bottom rows of Table 2. This repetition of the center run is necessary to estimate the population variance regardless of significant factors. Without the repetition of this center run or addition of fake factors^24^, the population variance has to be estimated by the residual sum of squares of the model containing significant parameters; hence, the estimator of the variance will not be unique but dependent on the chosen model.

**Table 2:** The design matrix with coded units (− 1, 0, 1), which correspond to the levels in Table 1.

Sample	Coded units of factors					
	*T*_crys_	*p*_oxy_	*T*_Dew_	Dewll time	Heating ramp	Rotation speed
1	0	1	1	1	1	1
2	0	− 1	− 1	− 1	− 1	− 1
3	1	0	1	1	1	1
4	− 1	0	− 1	− 1	− 1	− 1
5	1	1	0	1	1	1
6	− 1	− 1	0	− 1	− 1	− 1
7	1	1	1	0	1	1
8	− 1	− 1	− 1	0	− 1	− 1
9	1	1	1	1	0	1
10	− 1	− 1	− 1	− 1	0	− 1
11	1	1	1	1	1	0
12	− 1	− 1	− 1	− 1	− 1	0
13 (center run)	0	0	0	0	0	0
14 (center run)	0	0	0	0	0	0

The run in the bottom row (center run) was repeated to estimate the population variance regardless of significant factors.

After obtaining the experimental data, models are built following an appropriate model selection procedure. The data included in the model are the values of the property to be optimized, such as *J*_c_ at a certain magnetic field, a ratio of *J*_c_ at different fields and/or temperatures, or critical temperature *T*_c_. Although the model and its predictions depend on the selected property, we construct the model regarding ${\varvec{J}}_{{\mathbf{c}}}^{{{\mathbf{sf}}}}$ at 77 K in this work. The best second-order model (containing main, interaction, and quadratic effects) was selected among all the possible second-order models based on the Akaike information criterion with finite correction (AICc). AICc (or generally AIC) is an estimator to select a “good” model avoiding overfitting, which can explain the prediction values well. Supposing that the errors follow independent and identical normal distributions, AICc is expressed in the following equation for the least square estimation^23^.

1 $$ {\text{AICc}} = n\ln \left( {\hat{\sigma }^{2} } \right) + 2K + \frac{{2K\left( {K + 1} \right)}}{n - K - 1} $$

where *n* is the number of observations and $\hat{\sigma }^{2}$ is the estimator of the variance calculated by

2 $$ \hat{\sigma }^{2} = \frac{{\Sigma \left( {y_{i} - \widehat{{y_{i} }}} \right)^{2} }}{n} $$

where *y*_*i*_ and $\widehat{{y_{i} }}$ are observed and fitted value, respectively, of the *i*th factor.

## Results and discussion

Table 3 shows the ${\varvec{J}}_{{\mathbf{c}}}^{{{\mathbf{sf}}}}$ values at 77 K of Er-P and Er-A samples for the DSD experiment (Samples 1–14) together with pilot trials (Samples 15–30) that were obtained before starting the DSD experiment and used for confirmation of the equations (models) later. In the DSD experiment, the ${\varvec{J}}_{{\mathbf{c}}}^{{{\mathbf{sf}}}}$ values at 77 K ranged from 0 to 3.67 MA/cm^2^ for Er-P, and from 0 to 3.30 MA/cm^2^ for Er-A. Considering the measured thickness variation of about ± 30 nm (~ 10% of ErBCO layer), the *J*_c_ values also have an uncertainty of about ± 10%.

**Table 3:**
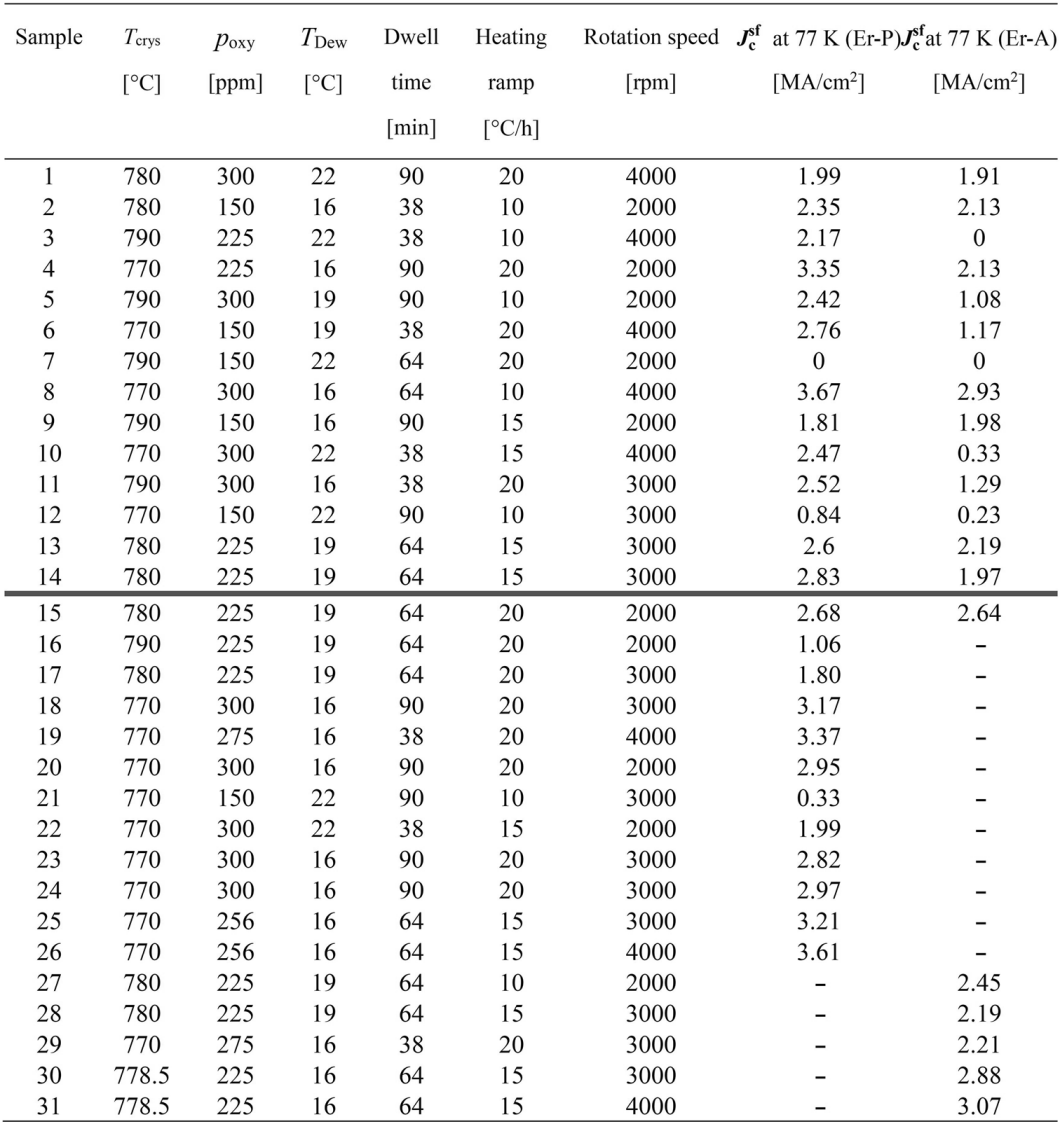
Measured ${\varvec{J}}_{{\mathbf{c}}}^{{{\mathbf{sf}}}}$ at 77 K of Er-P and Er-A. The latter twelve samples (below the bold line) are for the confirmation of the models [Eqs. (3) and (4)].

Among all the possible second-order models containing main, interaction, and quadratic effects, the models with minimum AICc, Eqs. (3) and (4), were selected for Er-P and Er-A respectively. Samples 1–14 were used to create these models.

3 $$ J_{{\text{c}}} \left( {\text{Er - P}} \right) = 2.737 - 0.417{{\left( {T_{{{\text{crys}}}} - 780} \right)} \mathord{\left/ {\vphantom {{\left( {T_{{{\text{crys}}}} - 780} \right)} {10}}} \right. \kern-\nulldelimiterspace} {10}} + 0.531{{\left( {p_{{{\text{oxy}}}} - 225} \right)} \mathord{\left/ {\vphantom {{\left( {p_{{{\text{oxy}}}} - 225} \right)} {75}}} \right. \kern-\nulldelimiterspace} {75}} - 0.623{{\left( {T_{{{\text{Dew}}}} - 19} \right)} \mathord{\left/ {\vphantom {{\left( {T_{{{\text{Dew}}}} - 19} \right)} 3}} \right. \kern-\nulldelimiterspace} 3} - 0.654\left\{ {{{\left( {p_{{{\text{oxy}}}} - 225} \right)} \mathord{\left/ {\vphantom {{\left( {p_{{{\text{oxy}}}} - 225} \right)} {75}}} \right. \kern-\nulldelimiterspace} {75}}} \right\}^{2} $$

4 $$ J_{{\text{c}}} \left( {\text{Er - A}} \right) = 2.049 - 0.303{{\left( {T_{{{\text{crys}}}} - 780} \right)} \mathord{\left/ {\vphantom {{\left( {T_{{{\text{crys}}}} - 780} \right)} {10}}} \right. \kern-\nulldelimiterspace} {10}} - 0.739{{\left( {T_{{{\text{Dew}}}} - 19} \right)} \mathord{\left/ {\vphantom {{\left( {T_{{{\text{Dew}}}} - 19} \right)} 3}} \right. \kern-\nulldelimiterspace} 3} - 0.876\left\{ {{{\left( {T_{{{\text{crys}}}} - 780} \right)} \mathord{\left/ {\vphantom {{\left( {T_{{{\text{crys}}}} - 780} \right)} {10}}} \right. \kern-\nulldelimiterspace} {10}}} \right\}^{2} $$

Table 4(a) and (b) list the important factors of these models for ${\varvec{J}}_{{\mathbf{c}}}^{{{\mathbf{sf}}}}$ at 77 K with *P* values of the coefficients (the smaller the *P* value is, the more likely the coefficient is not zero, hence, significant). The significant main factors for Er-P are *T*_crys_, *p*_oxy_, and *T*_Dew_, while for Er-A only *T*_crys_ and *T*_Dew_. Dwell time, heating ramp, and rotation speed are not significant for both sets of samples. The quadratic effect of *p*_oxy_ is significant for Er-P, and the quadratic effect of *T*_crys_ for Er-A. Neither of the two sets of samples showed any sign of two-factor interactions. Since at least some of the factors are usually correlated (e.g., the interaction between *T*_crys_ and *p*_oxy_ is certainly present^18^), we conclude that the investigated range in this work was not wide enough to detect such interactions.

**Table 4:** The statistical description of the model for *J*_c_(Er-P) and *J*_c_ (Er-A). The coefficients are expressed in the coded units. The tables describe only the significant terms with *P*-value^24^ smaller than 0.05.

Term	Coefficient	Standard error	*P* value
***J***_**c**_** (Er-P)**			
Intercept	2.737	0.221	< 0.001
*T*_crys_	− 0.417	0.140	0.015
*p*_oxy_	0.531	0.140	0.004
*T*_Dew_	− 0.623	0.140	0.002
(*p*_oxy_)^2^	− 0.654	0.261	0.034
***J***_**c**_** (Er-A)**			
Intercept	2.049	0.226	< 0.001
*T*_crys_	− 0.303	0.143	0.060
*T*_Dew_	− 0.739	0.143	< 0.001

**Figure 2:**
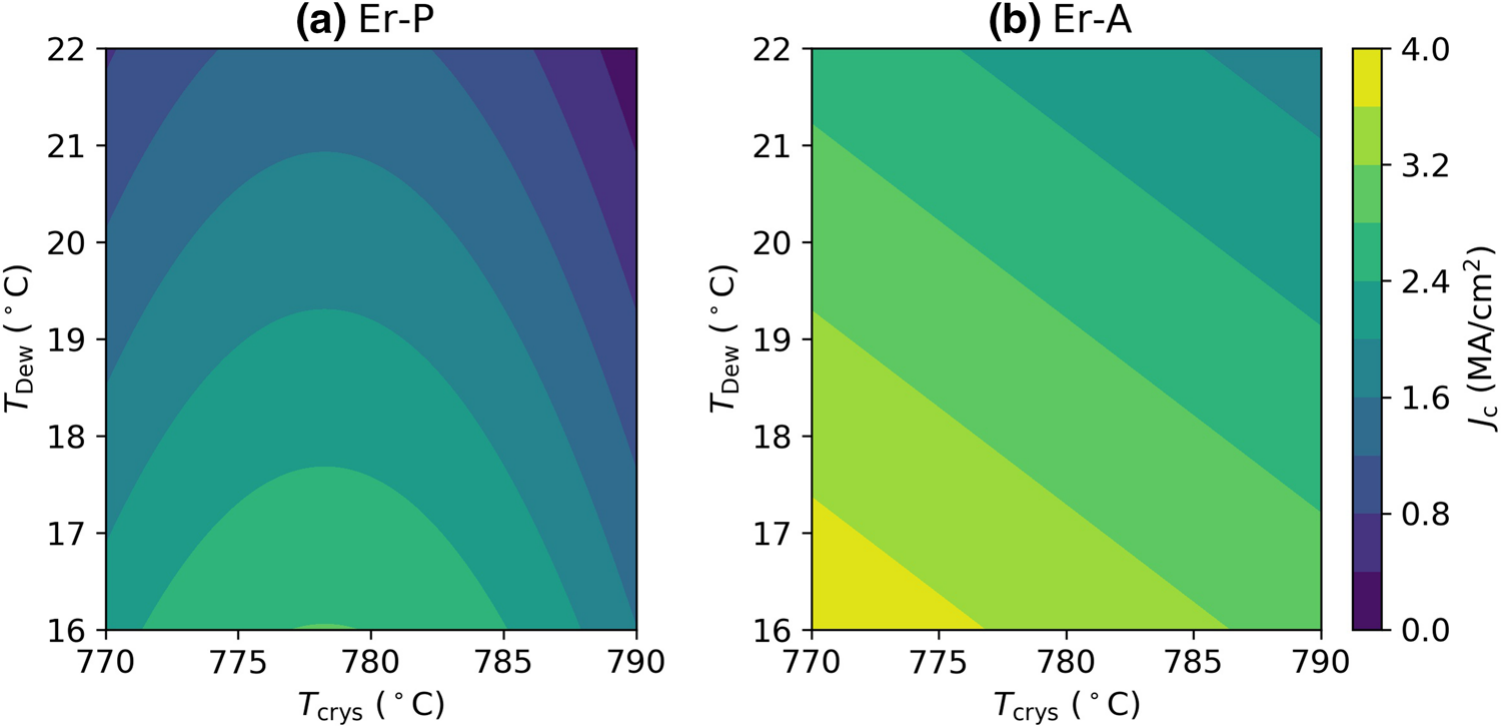
The behavior of (**a**) *J*_c_ (Er-P) and (**b**) *J*_c_ (Er-A) over humidity and *T*_crys_. In (**b**), *p*_**oxy**_ is fixed as the optimal level of 256 ppm (0.413 in coded unit) for *J*_c_ (Er-P).

Based on these models, Eqs. (3) and (4), the dependencies of *J*_c_ (Er-P) and *J*_c_ (Er-A) on *T*_Dew_ and *T*_crys_ are visualized in Fig. 2. Figure 2a shows that lower *T*_crys_ and lower *T*_Dew_ are crucial for improving *J*_c_ (Er-P) with the optimal *p*_oxy_ = 256 ppm (0.413 in coded unit), whereas Fig. 2b shows intermediate *T*_crys_ and lower *T*_Dew_ to be crucial for *J*_c_ (Er-A). The major differences between both sample types are that the desirable *T*_crys_ is lower for Er-P than for Er-A, and *p*_oxy_ is a significant parameter (in the investigated range) for Er-P but not for Er-A.

**Figure 3:**
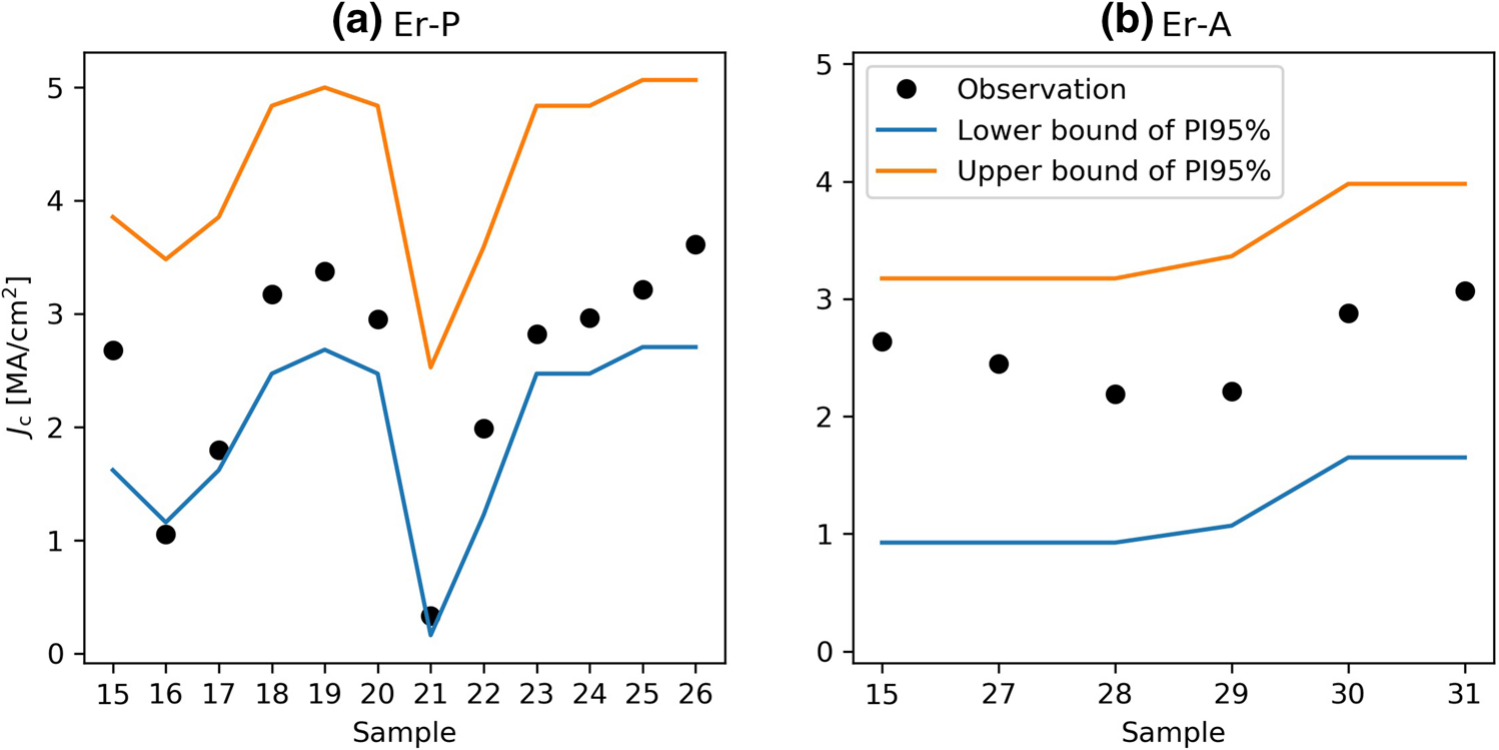
Model confirmation for (**a**) *J*_c_ (Er-P) and (**b**) *J*_c_ (Er-A). The conditions of the samples are described in Table 3. Samples 25 and 26 are optimal Er-P samples, Samples 30 and 31 are optimal Er-A samples. Note that measured *J*_c_ values have an uncertainty of about 10% because of the thickness uncertainty.

To confirm the validity of the *J*_c_ (Er-P) model Eq. (3), further twelve Er-P samples were selected (Samples 15–26 in Table 3) and their ${\varvec{J}}_{{\mathbf{c}}}^{{{\mathbf{sf}}}}$ values at 77 K measured. Figure 3a shows these *J*_c_ (Er-P) values together with their 95% prediction intervals (PI95%). Most of the data fall inside this prediction interval. Hence, the model is considered useful to predict *J*_c_ (Er-P). Furthermore, the model suggests that the maximum range of ${\varvec{J}}_{{\mathbf{c}}}^{{{\mathbf{sf}}}}$ at 77 K (2.9–4 MA/cm^2^) can be obtained with *T*_crys_ = 770 °C, *p*_oxy_ = 256 ppm, *T*_Dew_ = 16 °C (other parameters are arbitrary values). The samples made with these conditions are Sample 25 (3.2 MA/cm^2^) and Sample 26 (3.6 MA/cm^2^), which are indeed the highest level (considering 10% uncertainty of *J*_c_) and inside the prediction interval in Fig. 3a Similarly, the validation of *J*_c_ (Er-A) has been checked using Sample 15, and Samples 27–31 in Fig. 3b The optimal samples are Samples 30 and 31 prepared with *T*_crys_ = 778.5 °C and *T*_Dew_ = 16 °C (other parameters are not important). Samples 30 and 31 certainly outperformed the other Er-A samples.

Moreover, since not all the factors have quadratic effects, the global optimum seems to exist outside of the investigated range in this work. However, a one-factor-at-a-time approach for further improvement is acceptable because the interactions between the significant factors (*T*_crys_, *p*_oxy_, and *T*_Dew_) are not likely to be present near the investigated range.

## Conclusion

The crystallization process of ErBCO films deposited with CSD on SuperOx IBAD substrates was optimized via DSD, a novel design-of-experiment technique. This approach allowed investigating the effects of six crystallization parameters (*T*_crys_, *p*_oxy_, *T*_Dew_, dwell time, heating ramp, and rotation speed) with a considerably reduced number of trials compared to conventional one-factor-at-a-time approach. The crystallization was optimized regarding ${\varvec{J}}_{{\mathbf{c}}}^{{{\mathbf{sf}}}}$ at 77 K. Two types of ErBCO samples, Er-P and Er-A, prepared from the solutions containing Er-propionate and Er-acetate, respectively, were studied. Only 14 samples per sample type were necessary for this DSD experiment. The models based on the experiment reveal that *T*_crys_, *p*_oxy,_ and *T*_Dew_ are significant factors for *J*_c_ (Er-P), and only *T*_crys_ and *T*_Dew_ for *J*_c_ (Er-A) in the investigated range of the factors. As expected from the model for *J*_c_ (Er-P), a maximum of ~ 3.6 MA/cm^2^ was obtained with *T*_crys_ = 770 °C, *p*_oxy_ = 256 ppm, and *T*_Dew_ = 16 °C. Similarly, a maximum *J*_c_ (Er-A) of ~ 3.0 MA/cm^2^ was obtained with *T*_crys_ = 778.5 °C and *T*_Dew_ = 16 °C. Both models were confirmed by additional samples. These results indicate that DSD is a very attractive approach to optimize the properties of CSD-grown films. It could also be a powerful tool for the development of long-tape coated conductors as it could enormously reduce the effort in the optimization of the different process steps.
